# Target identification, screening and *in vivo* evaluation of pyrrolone-fused benzosuberene compounds against human epilepsy using Zebrafish model of pentylenetetrazol-induced seizures

**DOI:** 10.1038/s41598-019-44264-6

**Published:** 2019-05-27

**Authors:** Garima Tanwar, Arindam Ghosh Mazumder, Vijay Bhardwaj, Savita Kumari, Richa Bharti, Yamini, Damanpreet Singh, Pralay Das, Rituraj Purohit

**Affiliations:** 1 Structural Bioinformatics Laboratory, Biotechnology division, CSIR-Institute of Himalayan Bioresource Technology (CSIR-IHBT), Palampur, HP 176061 India; 2 Pharmacology and Toxicology Laboratory, CSIR-Institute of Himalayan Bioresource Technology, Palampur, 176061 Himachal Pradesh India; 3 Natural Product Chemistry and Process Development, CSIR-Institute of Himalayan Bioresource Technology, Palampur, India; 4 Academy of Scientific and Innovative Research (AcSIR), CSIR-Institute of Himalayan Bioresource Technology, Palampur, 176061 Himachal Pradesh India

**Keywords:** Natural products, Green chemistry

## Abstract

Pyrrolone-fused benzosuberene (PBS) compounds were semi-synthesized from *α,β,γ*-Himachalenes extracted from the essential oil of *Cedrus deodara* following amino-vinyl-bromide substituted benzosuberenes as intermediates. These PBSs compounds classified as an attractive source of therapeutics. The α-isoform of PI3K which is a pivotal modulator of PI3K/AKT/mTOR signaling pathway, responsible for neurological disorders like epilepsy, found as a potential target molecule against these 17 semi-synthesized PBS compounds using *in silico* ligand-based pharmacophore mapping and target screening. The compounds screened using binding affinities, ADMET properties, and toxicity that were accessed by *in silico* docking simulations and pharmacokinetics profiling. Ultimately two compounds *viz*., PBS-8 and PBS-9 were selected for further *in vivo* evaluation using a zebrafish (*Danio rerio*) model of pentylenetetrazol (PTZ)-induced clonic convulsions. Additionally, gene expression studies performed for the genes of the PI3K/AKT/mTOR pathway which further validated our results. In conclusion, these findings suggested that PBS-8 is a promising candidate that could bedeveloped as a potential antiepileptic.

## Introduction

Himachalenes a mixture of sesquiterpenes extracted from *Cedrus deodara* oil containing hexahydrobenzocycloheptene as basic skeleton can be easily converted to benzocycloheptene/benzosuberene, a core structure of several natural products like colchicine, allocolchicine, demethylsalvicanol and feveline etc. which are clinically reported bioactive compounds^1,2^. In earlier developments, *α*, *β*, *γ*-himachalenes as a mixture were applied through sequential and consecutive approaches for the synthesis of substituted benzosuberenes as a reactive and bio-active precursor^3,4^. The present study aimed to search target molecules from benzosuberene classes of compounds which could serve as promising therapeutics for treating the target disease. After successful *in*-*silico* ligand-based pharmacophore mapping and target identification^5^, we found phosphoinositide-3-kinase-α (PI3K-α) as a potential target against the selected molecules. PI3Ks are lipid kinases that control mTOR (mammalian target of rapamycin) signaling pathway which is responsible for cell proliferation, cell invasion, cell migration and cell death^6^. The mTOR pathway is a frequent target of epilepsy treatment. mTOR hyperactivation has been found to be active in many types of human cancers and neurological disorders. mTOR is a serine/threonine protein kinase that belongs to the PI3K family and is encoded by the MTOR gene^7,8^. PI3K consist of three classes: Class I, Class II and Class III, in which Class I is divided into Class IA and Class IB. PI3K-α falls under the Class IA. It catalyze the phosphorylation of 3′-hydroxyl group of the inositol ring of phosphatidylinositol and also activated by cell surface receptors such as receptor tyrosine kinases (RTKs), G-protein coupled receptors (GPCRs) and small G-protein oncogenes (Ras)^9,10^. They are heterodimers of catalytic and regulatory subunits, such as p110 (catalytic) and p85 (regulatory)^11,12^. Human cells contain the PIK3CA gene that encodes catalytic subunit such as p110α of class I PI3K^13^. Phosphorylation of tyrosine kinase receptor results in the activation of PI3K which activates cascading steps of phosphorylation. PI3K further activates AKT, which in turn, phosphorylates mTOR, that has downstream regulatory effects on genes such as ribosomal protein S6 kinase (*Rps6kb1*) and ribosomal protein S6 (*Rps6*). PI3K inhibition counteracts the downstream activation of mTOR^14,15^. PI3K/AKT/mTOR signaling pathway has been proven to be involved in neurogenesis and dysregulation, or hyperactivation of this signaling pathway strongly associated with many severe brain disorders, including epilepsy^16^. Moreover, mutations in this signaling pathway have been found in a large number of human patients with epilepsy^17^.

Epilepsy is the fourth most common neurological disorder that affects people of all ages. It is characterised by recurrent neuronal seizures with or without loss of consciousness^15^. If not treated well, it might result in brain damage which could even lead to death^18^. Many anti-epileptic drugs are available in the market which possess significant adverse effects on the health of the individual^19^. This necessitates an urgent need for an effective therapeutic drug against epilepsy with minimal or no side effects.

**Figure 1:**
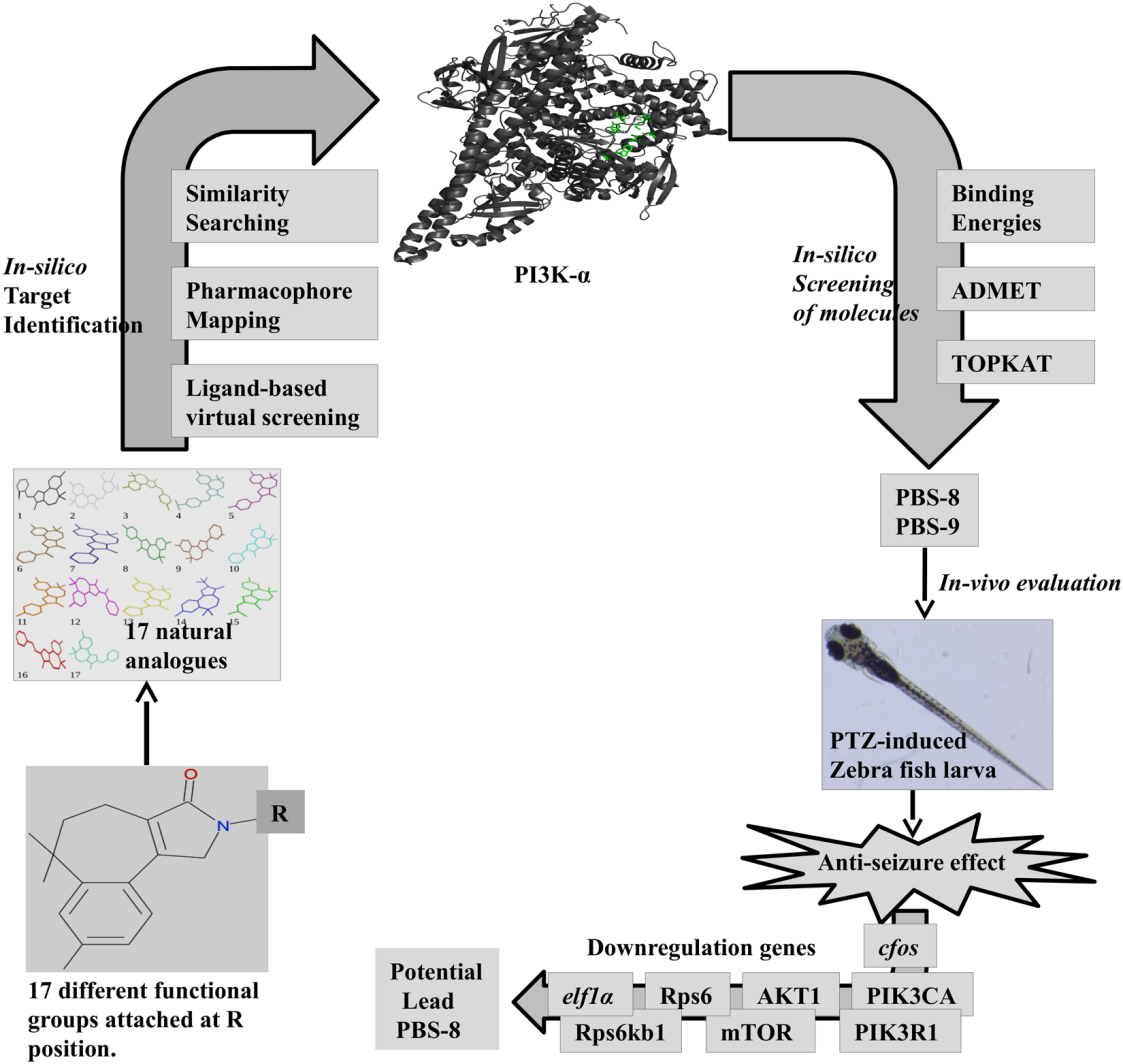
Overview of computational and experimental analysis of potential lead compounds.

These demands initiated an enormous interest in screening the active therapeutic agents for the treatment of epilepsy. Our present study suggested a potential lead compound that could be used to treat epilepsy by compressing PI3K-α activation. In this direction, we screened and evaluated benzosuberene classes of compounds by *in silico* approaches. Further, to validate  the activity of the computationally suggested compound(s) against epilepsy, we tested these compounds in a Zebrafish (*Danio rerio*) larvae model of pentylenetetrazol (PTZ)-induced convulsions. The study identified a potential lead compound against epilepsy, and this workflow is presented in Fig. 1. As this compound is naturally derived, therefore it could probably be a better candidate for the therapeutic purpose with more bio- compatibility and less toxicity.

## Results

### Semi-synthesis of PBS compounds

**Figure 2:**
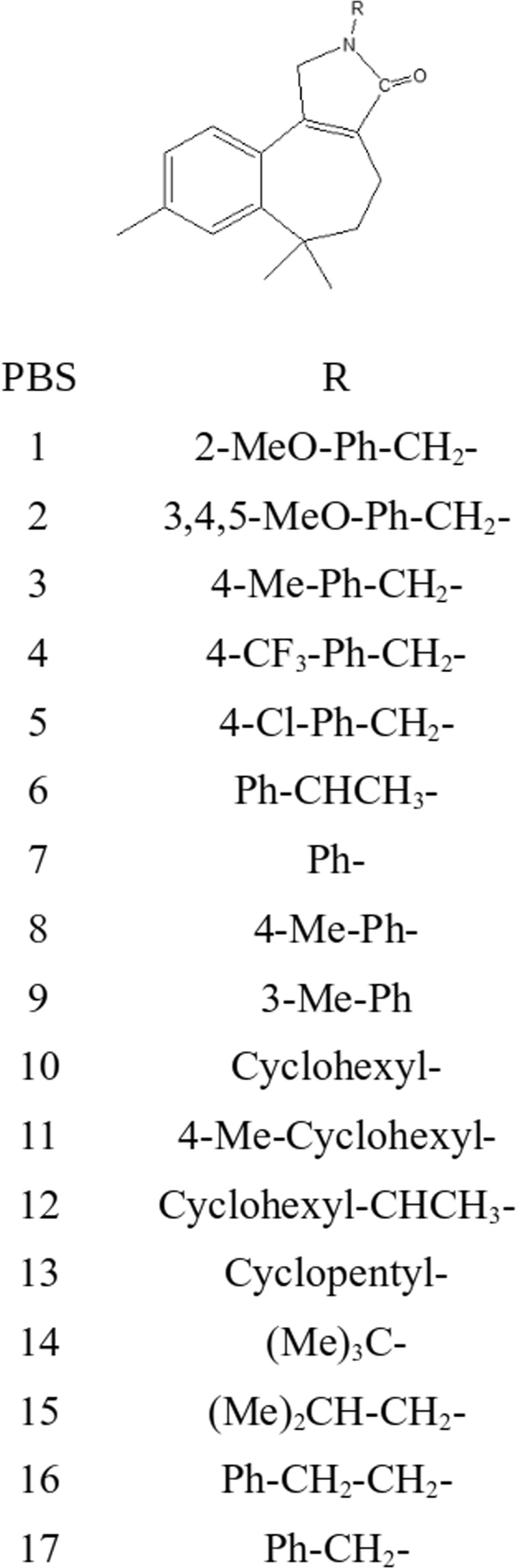
Pyrrolone-fused benzosuberenes (1–17 molecules) with different functional groups.

The pyrrolone fused benzosuberenes (PBSs) used under this study were semi-synthesized from *α,β,γ*-himachalenes following the intermediates of amino-vinyl-bromide substituted benzosuberenes. Further, these intermediates in the presence of oxalic acid as *in situ* CO source under palladium catalyzed condition gave pyrrolone-fused benzosuberenes (PBSs) (Fig. 2, ligand 1–17). Under this study, several functional groups were found to be toleratnt and ended with good yields^20^.

### Identification of a target molecule

Further, to identify the target molecule against 17 PBS compounds, we used a ligand-based virtual screening approach^21^ with the help of Accelrys Discovery studio package. The 3D pharmacophore model against these PBS ligands were mapped using the interaction pattern of cations, anions, aromatic, aliphatic, hydrophobic and hydrogen bond donors/acceptors^5^. The pharmacophore model thus generated was then used to search the pre-existing structured databases to identify the molecular structure that best matches with the pattern of that pharmacophore map. This similarity search unearths PI3K (α-isoform) as the biological target against our PBS compounds.

### Analyses of binding energies and binding interactions

**Figure 3:**
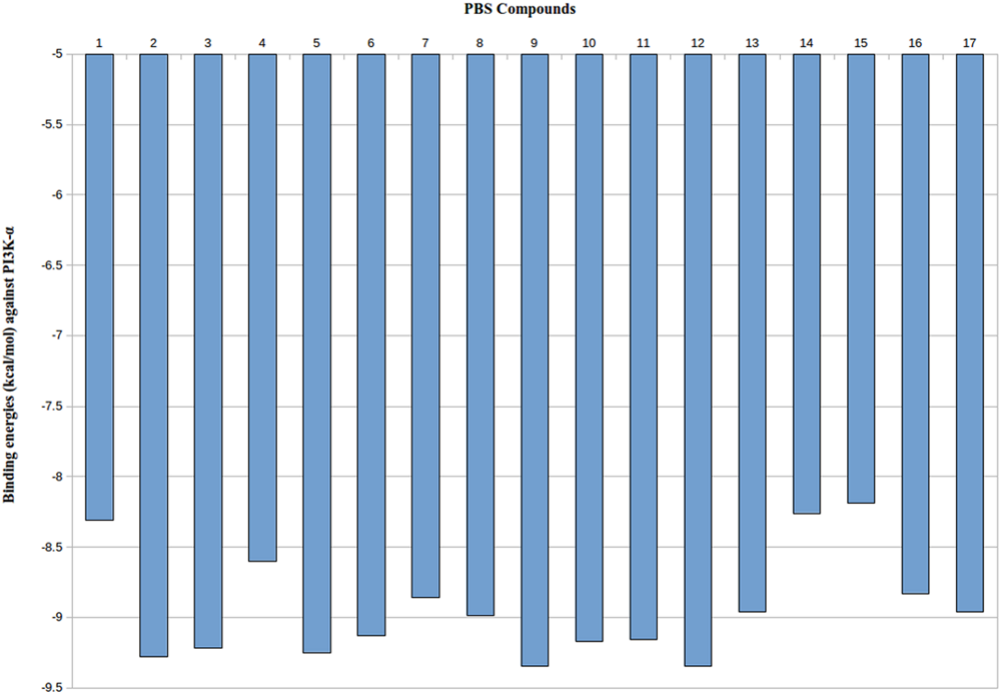
A histogram is showing binding energies obtained by Autodock 4.2.6 docking results. 1 to 17 PBS compounds are illustrated in the x-axis of the graph; binding energies are illustrated in -y-axis of the graph.

**Figure 4:**
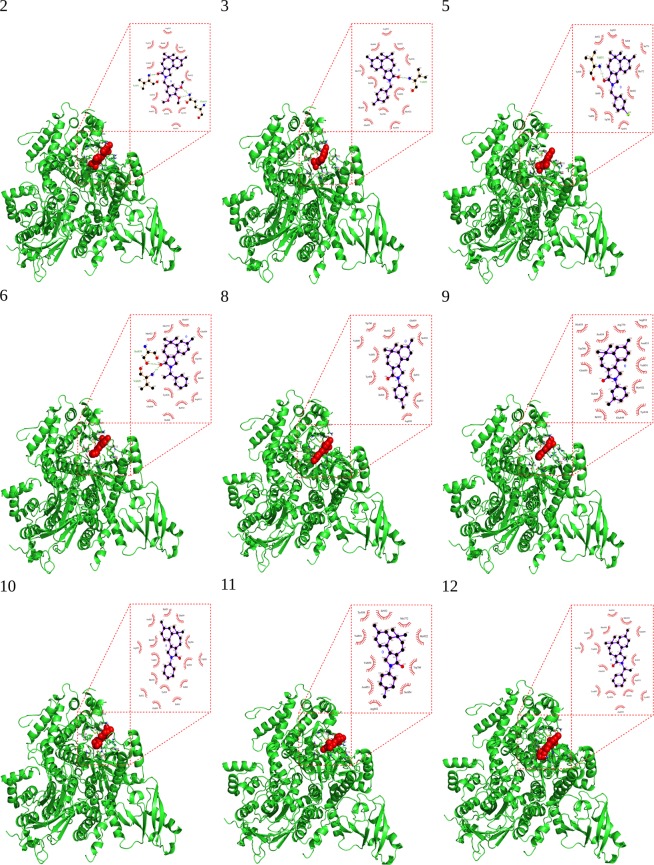
Docked conformations of selected PBS compounds (represented as red color spheres) complexed with PI3K-α (represented as a green color cartoon); that are screened through binding energies obtained by Autodock 4.2.6. An enlarged view of docked PBS ligands and PI3K-α residues shows 2D interactions using LigPlot^+^. Purple lines represent ligand bonds, and yellow lines represent non-ligand bonds. Hydrogen-bonds are represented by green dotted lines and distances between atoms are expressed in Å. Residues involved in hydrophobic interactions are identified by a red color semicircle surrounding them with corresponding atoms represented by black dots.

For enumeration of specific inhibitors against α isoform of PI3K lipid kinase, we docked our 17 naturally originated compounds with this isoform. We calculated the energy of interaction between PI3K-α and 17 PBS ligands. Docking with Autodock 4.2.6^22^ exhibited different binding energies of 17 docked ligands with PI3K, ranging from −8 to −10 kcal/mol (Fig. 3). Lowest binding energies of our 17 PBS compounds docked with α isoform following the ligand order of PBS-9, PBS-12 (−9.35 kcal/mol) < PBS-2 (−9.28 kcal/mol) < PBS-5 (−9.25 kcal/mol) < PBS-3 (−9.22 kcal/mol) < PBS-10 (−9.17 kcal/mol) < PBS-11 (−9.16 kcal/mol) < PBS-6 (−9.13 v) < PBS-8 (−8.99 kcal/mol) < PBS-13, PBS-17 (−8.96 kcal/mol) < PBS-7 (−8.86 kcal/mol) < PBS-16 (−8.83 kcal/mol) < PBS-4 (−8.60 kcal/mol) < PBS-1 (−8.31 kcal/mol) < PBS-14 (−8.26 kcal/mol) < PBS-15 (−8.19 kcal/mol), as shown in Table 1. The atomic interactions were further explored by LigPlot^+^ v.1.4 software^23^. This software could plot 2D views of in-depth ligand bonds, non-ligand bonds, hydrogen bonding and hydrophobic interactions pattern between the docked ligands and the active site residues of the corresponding receptor (Fig. 4).

**Table 1:** Docking results of 17 PBS compounds with α isoform of PI3K lipid kinase by using Autodock 4.2.6 software.

PBS compounds	Estimated free energy of binding (kcal/mol)	Estimated inhibition constant (nM)	Final inter-molecular energy (kcal/mol)	VdW + Hbond + desolvation energy (kcal/mol)	Electrostatic energy (kcal/mol)	Final total internal energy (kcal/mol)	Torsional free energy (kcal/mol)	Unbound system’s energy (kcal/mol)
1	−8.72	404.98	−9.62	−9.61	−0.01	−0.68	0.89	−0.68
2	**−9.28**	157.26	−10.77	−10.68	−0.09	−0.67	1.49	−0.67
**3**	**−9.22**	175.81	−9.81	−9.79	−0.02	−0.33	**0.60**	−0.33
4	−8.60	493.26	−9.50	−9.54	0.04	−0.47	0.89	−0.47
**5**	**−9.25**	164.66	−9.85	−9.82	−0.03	−0.29	**0.60**	−0.29
**6**	**−9.13**	204.60	−9.72	−9.69	−0.03	−0.47	**0.60**	−0.47
7	−8.86	319.12	−9.16	−9.15	−0.01	−0.41	0.30	−0.41
**8**	**−8.99**	257.31	−9.29	−9.28	−0.01	−0.33	**0.30**	−0.33
**9**	**−9.35**	140.68	−9.65	−9.64	−0.00	−0.37	**0.30**	−0.37
**10**	**−9.17**	189.60	−9.47	−9.47	−0.00	−0.44	**0.30**	−0.44
**11**	**−9.16**	194.27	−9.45	−9.41	−0.04	−0.47	**0.30**	−0.47
**12**	**−9.35**	140.70	−9.94	−9.96	0.02	−0.60	**0.60**	−0.60
13	−8.96	271.91	−9.26	−9.25	−0.01	−0.18	0.30	−0.18
14	−8.26	879.83	−8.56	−8.60	0.04	−0.34	0.30	−0.34
15	−8.19	997.31	−8.78	−8.78	−0.00	−0.36	0.60	−0.36
16	−8.83	338.11	−9.72	−9.67	−0.06	−0.43	0.89	−0.43
17	−8.96	270.93	−9.56	−9.57	0.02	−0.24	0.60	−0.24

Estimated free energy of binding = (Final intermolecular energy + final total internal energy + Torsional free enrgy – Unbound system’s free energy).

### Analyses of drug-likeliness

To determine the drug-likeliness of our PBSs compounds, we calculated their ADMET properties. The screened results of ADMET were summarized in Table 2, revealing six descriptors such as absorption, solubility, cytochrome P_450_ 2D6 (CYP2D6), plasma protein binding (PPB), hepatotoxicity, and AlogP98. Easy absorption of the drug through blood brain barrier (BBB) measured by its AlogP98 value which must be less than 5. The obtained absorption levels determine drug absorption and absorption decreases inversely with the level, i.e., level 0 denotes proper absorption, level 1 denotes moderate absorption and so on. To determine the inhibitory effect and toxicity of the drug CYP2D6 and hepatotoxicity descriptors gave two predicted classes, such as class 0 (non-inhibitor and non-toxic) and class 1 (inhibitor and toxic). Another ADMET descriptor PPB has given two results true or false that symbolizes the binding or non-binding of the drug. Moreover, solubility descriptor predicted the molar solubility of the drug, which gives good molar solubility within a range of −2.0 and −4.0. If it is below that range, solubility decreases gradually and becomes extremely low below −8.0, and above that range, it increases gradually to become too soluble at 0.0.

**Table 2:** ADMET descriptors. AlogP98 must be less than 5 for good absorption through BBB.

PBS compounds	ADMET Absorption level	ADMET CYP2D6	ADMET Hepatotoxicity	ADMET PPB	ADMET Solubility	AlogP98
1	0	1	0	True	−6.454	4.953
2	0	0	1	True	−6.253	4.921
**3**	**0**	**0**	**0**	True	−6.969	5.456
4	1	1	0	True	−7.444	5.912
5	**0**	1	1	True	−7.125	5.634
**6**	**0**	**0**	**0**	True	−6.909	5.347
7	0	0	0	True	−6.606	4.963
**8**	**0**	**0**	**0**	True	−7.081	5.449
**9**	**0**	**0**	**0**	True	−7.090	5.449
**10**	**0**	**0**	**0**	True	−6.852	5.245
**11**	**0**	**0**	**0**	True	−7.123	5.496
12	1	0	0	True	−7.437	5.953
13	0	0	0	True	−6.438	4.788
14	0	0	0	True	−5.963	4.318
15	0	0	0	True	−5.988	4.578
16	0	0	0	True	−6.666	5.291
17	0	0	0	True	−6.505	4.970

Absorption of drug was determined by obtained levels: 0(good), 1(moderate), 2(low), 3(very low). Hepatoxicity determines toxicity of drug by predicted classes: 0(non-toxic) and 1(toxic). CYP2D6 descriptor determines inhibitory effect by predicted classes: 0(non-inhibitor) and 1(inhibitor). PPB (plasma protein binding) determines binding of drug, true symbolizes binding and false symbolizes non-binding. ADMET solubility descriptor predicts molar solubility of drugs within the ranges: −6.0 to −4.0 (low solubility), −4.0 to −2.0 (good solubility), and −2.0 to 0.0 (optimal solubilty).

**Figure 5:**
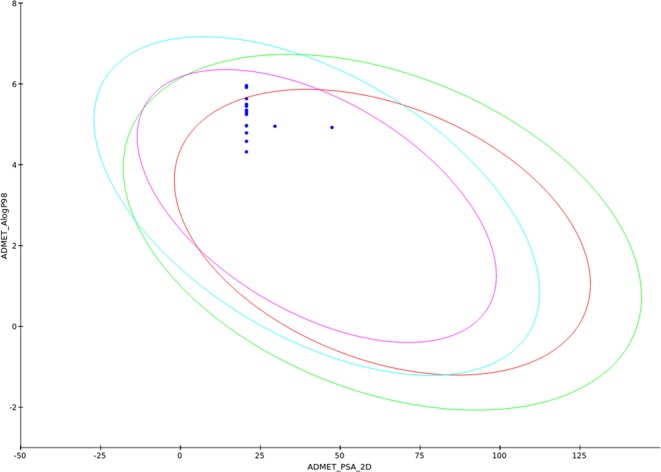
A plot of AlogP98 versus 2D Polar Surface Area (PSA) for our PBS compounds. The plot is showing green and blue colored eclipses of 99% confidence limits for intestinal absorption and blood-brain barrier (BBB), as well as red and pink colored eclipses of 95% confidence limits for intestinal absorption and BBB.

ADMET model also developed using two descriptors such as 2D polar surface area (2D-PSA) and AlogP98 (Fig. 5). This model includes the eclipses of 99% and 95% confidence limits which were used to define the regions with compounds having proper intestinal absorption and penetration across the BBB. The results of the obtained ADMET model showed that all our PBS compounds fell inside all the eclipses expect two of them fell outside the eclipse of 95% confidence limits for intestinal absorption.

Another method used to determine the therapeutic compatibility of the drug is toxicity prediction by komputer assisted technology (TOPKAT), summarized in Table 3. TOPKAT is a useful tool for in-silico prediction of toxicity quantitatively, and it is employed in quantitative structure-activity relationship (QSTR) models. Moreover, with the help of these QSTR models, it calculates probability values and evaluates toxicity through them. It follows the criterion of checking the components in the optimal predictive space (OPS), and when they lie outside then the results were considered as unreliable, i.e., false positives. Obtained Ames probability values determine the level of toxicity, such as, 0.0 to 0.30 (non-toxic), 0.30 to 0.70 (inter vocal), and 0.70 to 1.0 (toxic). Other additional descriptors provided was Ames mutagenicity, Ames enrichment and Ames score to determine the reliability of the predictions. It also provides values of the carcinogenic potency of TD50 mouse, rat oral LD50, rat inhalational LC50, and Daphina EC50. Increase in TD50, LD50, LC50, and EC50 values predicts the decrease in toxicity and increase in safety index of the drug which makes it more potent.

**Table 3:** TOPKAT (Toxicity Prediction by Komputer-Assisted Technology) results of our 17 PBS compounds using Biovia Discovery Studio.

PBS Compounds	TOPKAT Carcinogenic potency of TD50 mouse	TOPKAT Ames mutagenicity	TOPKAT Ames applicability	TOPKAT Ames probability	TOPKAT Ames enrichment	TOPKAT Ames score	TOPKAT Rat oral LD50 (g/kg body weight)	TOPKAT Rat oral LD50 applicability	TOPKAT Rat inhalational LC50 (mg/m3/h)	TOPKAT Rat inhalational LC50 applicability	TOPKAT Daphina EC50 (mg/l)	TOPKAT Daphina EC50 applicability
1	151.984	Non-mutagen	OPS are in range	0.441585	0.790843	−8.80134	11.4371	OPS are in range	14707.7	Out of range	0.303517	OPS are in range
2	47.1358	Non-mutagen	OPS are in range	0.197512	0.353729	−14.215	12.5095	OPS are in range	7855.16	Out of range	0.178047	OPS are in range
3	22.3026	Non-mutagen	OPS are in range	0.484231	0.867221	−7.86869	26.6463	OPS are in range	18673	OPS are in range	0.439018	OPS are in range
4	31.5215	Non-mutagen	OPS are in range	0.136184	0.243894	−15.9872	7.74428	OPS are in range	70998.5	Out of range	0.258939	OPS are in range
5	18.2656	Non-mutagen	OPS are in range	0.343058	0.61439	−10.8861	13.8323	OPS are in range	13463.7	Out of range	0.404508	OPS are in range
6	**105.955**	Non-mutagen	OPS are in range	0.409372	0.733153	−9.48765	9.18902	OPS are in range	42313.4	Out of range	1.42888	Out of range
7	120.698	Non-mutagen	OPS are in range	0.420443	0.75298	−9.25297	6.50877	OPS are in range	19340.9	OPS are in range	0.860618	Out of range
**8**	**46.7186**	Non-mutagen	OPS are in range	0.454854	0.814608	−8.51479	8.39242	OPS are in range	19615.1	OPS are in range	1.06299	OPS are in range
**9**	**73.8733**	Non-mutagen	OPS are in range	0.437366	0.783289	−8.89188	13.986	OPS are in range	18618.4	OPS are in range	0.869266	Out of range
10	**45.17**	Non-mutagen	OPS are in range	0.362803	0.649753	−10.469	5.30094	Out of range	16278.5	OPS are in range	1.20416	Out of range
11	41.7067	Non-mutagen	OPS are in range	0.309891	0.554991	−11.5949	9.04459	OPS are in range	15305.7	OPS are in range	1.10545	Out of range
12	36.7957	Non-mutagen	OPS are in range	0.151816	0.271891	−15.4947	7.20874	OPS are in range	36693.2	OPS are in range	0.753192	Out of range
13	51.157	Non-mutagen	OPS are in range	0.426716	0.753469	−9.24717	5.86107	Out of range	16939.3	OPS are in range	1.34943	Out of range
14	194.553	Non-mutagen	OPS are in range	0.504195	0.902975	−7.41779	12.7876	OPS are in range	19548	OPS are in range	1.99138	OPS are in range
15	76.8609	Non-mutagen	OPS are in range	0.459165	0.822329	−8.42106	7.63858	OPS are in range	45816	OPS are in range	2.83605	OPS are in range
16	60.9684	Non-mutagen	OPS are in range	0.28406	0.50873	−12.1594	8.72366	OPS are in range	15912.3	OPS are in range	0.418196	Out of range
17	57.7226	Non-mutagen	OPS are in range	0.523396	0.937361	−6.97266	12.3785	OPS are in range	18444.9	OPS are in range	0.593537	Out of range

Further analyses of drug-likeliness were performed by Lipinski’s rule-of-five to determine the ability of the drug to diffuse passively through the BBB. Lipinski’s rule-of-five follows the criteria of number of violations listed in Table 4 such as, molecular weight of compound should be less than 500, AlogP value should be less than 5, hydrogen bond donors should be less than 5, hydrogen bond acceptors should be less than 10, and number of rotatable bonds should be less than 5, respectively.

**Table 4:** Physicochemical properties of PBS compounds on the basis of Lipinski’s rule-of-five.

PBS compounds	Num_H_Acceptors_lipinski	Num_H_Donors_lipinski	Molecular weight	AlogP	Num_rotatable bonds	Molecular polar surface area	Num_H_acceptors	Num_H_donors
1	3	0	361.477	5	3	29.54	2	0
2	5	0	421.529	4.9	5	48	4	0
3	2	0	345.477	5.5	2	20.31	1	0
4	2	0	399.449	5.9	3	20.31	1	0
5	2	0	365.896	5.6	2	20.31	1	0
6	2	0	345.477	5.3	2	20.31	1	0
7	2	0	317.424	5	1	20.31	1	0
8	2	0	331.451	5.4	1	20.31	1	0
9	2	0	331.451	5.4	1	20.31	1	0
10	2	0	323.472	5.2	1	20.31	1	0
11	2	0	337.498	5.5	1	20.31	1	0
12	2	0	351.525	6	2	20.31	1	0
13	2	0	309.445	4.8	1	20.31	1	0
14	2	0	297.434	4.3	1	20.31	1	0
15	2	0	297.434	4.6	2	20.31	1	0
16	2	0	345.477	5.3	3	20.31	1	0
17	2	0	331.451	5	2	20.31	1	0

### Effect on PTZ-induced clonic seizures

**Figure 6:**
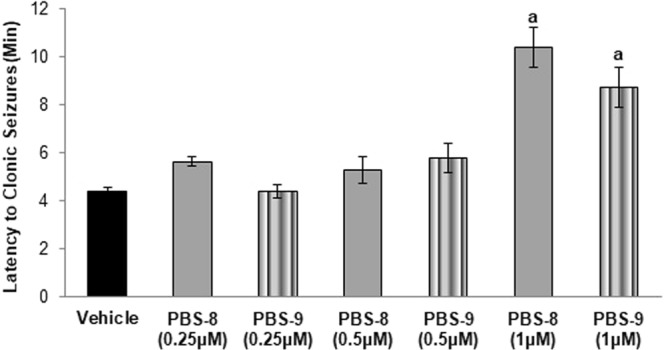
Effect of PBS-8 and PBS-9 on the latency to PTZ-induced clonic-like seizures. ^a^P < 0.05 as compared to vehicle control group.

The exposure with 8 mM PTZ showed the appearance of clonic seizure in vehicle control larvae with a latency of 4.42 ± 0.15 min. Pre-incubation with 1 µM concentration of PBS-9 (P = 0.002) and PBS-8 (P < 0.001) resulted in a marked increase in latency to clonic-like seizures in comparison to vehicle control. However insignificant difference in latency to first clonic-like seizure was observed at 1 µM concentration of PBS-9 and PBS-8 (P = 0.670). The group pre-incubated with sodium valproate showed a significant (P < 0.001) increase in latency to clonic-like seizures. The effect of both PBS-8 (P = 1.00) and PBS-9 (P = 0.639) at 1 µM concentration was found to be equipotent when compared to sodium valproate. Both test compounds were found to be ineffective at 0.25 µM and 0.5 µM concentrations as compared to vehicle control (Fig. 6).

### Effect on mRNA levels

**Figure 7:**
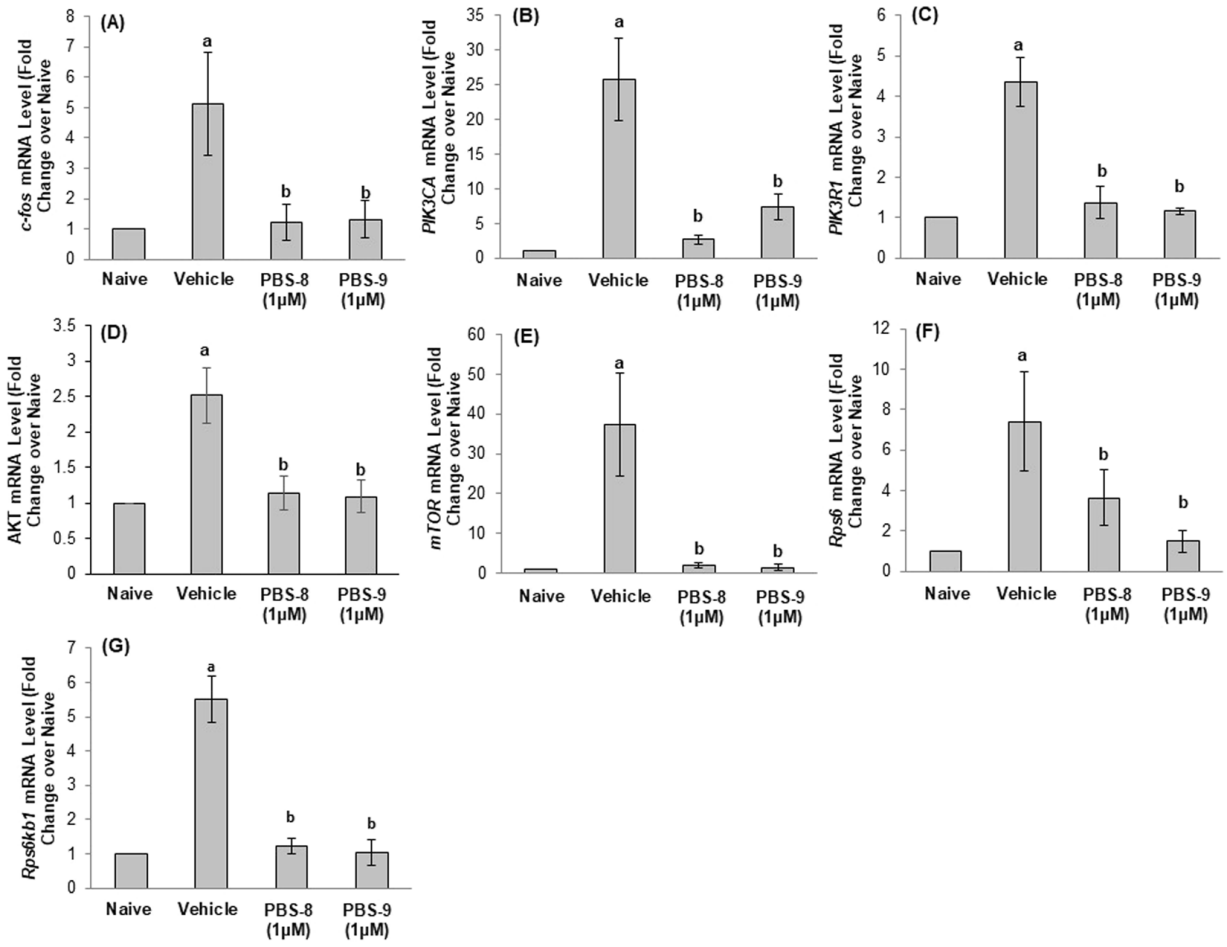
Effect of PBS-8 and PBS-9 on mRNA expression of *c-fos* (**A**), *PIK3CA* (**B**), *PIK3R1* (**C**), *AKT* (**D**), *mTOR* (**E**), *Rps6* (**F**) and *Rps6kb1* (**G**) in the zebrafish larvae exposed to PTZ. ^a^P < 0.05 as compared to naïve group; ^b^P < 0.05 as compared to vehicle control group.

Larvae exposure to PTZ resulted in a significant increase in mRNA levels of *c-fos* (P = 0.003), *PIK3CA* (P < 0.001), *PIK3R1* (P < 0.001), *AKT1* (P < 0.001), *mTOR* (P < 0.001), *Rps6* (P 0.003) and *Rps6kb1* (P < 0.001) as compared to naive. The level of *c-fos* mRNA was found to be significantly decreased in PBS-8 (P = 0.004), and PBS-9 (P = 0.005) exposed larvae in contrast to vehicle control. Furthermore, pre-incubation with a 1 µM concentration of PBS-8 and PBS-9 showed a significant (P < 0.001) decrease in mRNA levels of *AKT, PIK3CA, PIK3R1, mTOR*, and *Rps6kb1* as compared to the vehicle control larvae. A marked decrease in mRNA level of *Rps6* was also observed in the larvae exposed to PBS-8 (P = 0.049) and PBS-9 (P = 0.005) and as that of vehicle control (Fig. 7).

## Discussion

For identification of the biological target against ligand 1–17, they were undergone through *in silico* studies. The procedures of virtual screening were performed to identify the biological target using Discovery studio package. Virtual screening is an efficient approach that is widely used for the discovery of novel compounds^24^. In the absence of target molecule information ligand-based virtual screening approach had been successfully applied, such as pharmacophore mapping and similarity searching^5^.

PI3K-α was a resulted target of ligand-based virtual screening against these 17 PBSs compounds. PI3K as reported plays an essential role in the activation of mTOR signaling pathway. The role of the PI3K/AKT/mTOR pathway has been widely deciphered in epilepsy^14,25^. Studies suggested that injury due to seizures led to activation of the pathway which further propagates seizure progression and related pathogenic changes^26^. Furthermore, the constitutive stimulation of the pathway has been well explored in various *in vitro*^27^ and *in vivo* models^28^. Studies conducted on PI3K suggested its phosphatidylinositol group to be responsible for the emergence of a large number of mitotic factors, thus resulting in the proliferation of cells^29^ through phosphorylating AKT and activation of its downstream genes such as mTOR, *Rps6*, and *Rps6kb1*. It has been found that all mammalian cells when activated by receptor tyrosine kinases, expresses at least one of the isoforms of PI3K. Moreover, the PI3K (class IA) is functional when the catalytic subunit p110α binds with its regulatory adapter protein p85α to form a dimer^30^. Thus this target is beneficial for the development of new treatment avenues in epilepsy.

In this study, *in silico* docking and pharmacokinetic profiling were performed for screening of more drug-likely PBSs ligands against the PI3K-α protein. Molecular docking is a computational approach widely used in the drug discovery process^31,32^. These PBSs compounds were then screened by obtaining binding energies using docking simulations. Smaller the binding energy, more potential it is. Based on binding energies nine compounds were screened viz., PBS-2, PBS-3, PBS-5, PBS-6, PBS-8, PBS-9, PBS-10, PBS-11 and PBS-12, and based on the torsional free energy they were screened down to eight viz., PBS-3, PBS-5, PBS-6, PBS-8, PBS-9, PBS-10, PBS-11, and PBS-12. Consequently, the screening of these remaining eight PBS compounds were done using their ADMET properties. PBS-12 was screened out based on ADMET adsorption level descriptor as well as PBS-5 was screened out based on its inhibition effect and toxicity showed by CYP2D6 and hepatotoxicity descriptors. After ADMET screening six compounds were selected viz., PBS-3, PBS-6, PBS-8, PBS-9, PBS-10, and PBS-11. Further screening of PBSs compounds were done based on TOPKAT results. PBS-3 and PBS-11 compounds were screened out based on their low potency values obtained by TOPKAT carcinogenic potency of TD50 mouse descriptor. Moreover, PBS-6 and PBS-10 were screened out based on the applicability of rat oral LD50, rat inhalational LC50, and Daphina EC50. Finally,*in silico* docking studies and pharmacokinetic profiling suggested that PBS-8 and PBS-9 compounds were found suitable inhibitor for PI3K-α. Finally, they were evaluated through *in vivo* studies using zebrafish as a model organism for human epilepsy.

Since the past few decades, zebrafish has gained popularity as a developed disease model. The foremost criterion for using zebrafish in epilepsy research is to ascertain seizure like clonic convulsions as depicted by the Racine scale in rodent model^33^. Accordingly, in the present study, seizure-like behavior in 7 dpf (days post fertilization) zebrafish larvae were established using PTZ, and latency to first clonic seizure was recorded. The study found that, exposure to the test compounds (PBS-8 and PBS-9) depicted a considerable increase in PTZ-mediated clonic seizure latency at 1 µM concentration. Epileptic studies conducted on rodent models have revealed that seizure generation leads to immediate early genes expression, particularly *c-fos* upregulation in the brain^34^, a similar observation has also been made earlier in the zebrafish larvae model^33^. In line with this observation, the present study showed increased *c-fos* expression in PTZ exposed vehicle control larvae, and subsequent decrease upon pre-incubation with PBS-8 and PBS-9, supporting its anti seizure effect. Our findings were further supported by previous work showing *c-fos* downregulation in larvae treated with an antiseizure compound^35^.

Our study revealed that the genes encoding these units, *i.e*., p110α (*PIK3CA* gene) and p85α (*PIK3R1* gene) were upregulated in the larvae exposed to PTZ. Pre-incubation with PBS-8 and PBS-9 showed a reduction in their expression. The fact that the intricate mechanism of PI3K/AKT activation leading to mTOR hyperactivation in rodent models of epilepsy^36^ can be corroborated with our findings, which showed that the expression of all the downstream genes, *i.e.,AKT*, *mTOR*, *Rps6*, and *Rps6kb1* were reduced dramatically following drug treatment on PTZ treated larvae.

The study identified few potential lead compounds against epilepsy. Our adopted approaches addresse the complexity in searching enormous natural bioactive space. Moreover cellular targets of a natural lead is crucial for the process of drug discovery. We strongly recommand computational exploration in target identification and screening of lead before going for an *in-vivo* analysis. It helps to reduce the effort and time of a researcher.

## Materials and Methods

### Synthesis of chemical compounds

All the 1–17 pyrrolone-fused benzosuberene (PBS) compounds used under this study were synthesized following our earlier developed protocols^20^. These compounds (ligand 1–17) were synthesized with different functional groups at the specific position denoted by R, as shown in Fig. 2. Further, these molecules were investigated for therapeutic applications.

### Ligand-based Virtual screening to natural analogs

Accelrys Discovery studio package (Dassault Systèmes BIOVIA, 2017R2, San Diego) was used for deriving pharmacophore mapping^5^, that is a type of ligand-based virtual screening. Naturally extracted seventeen ligands were fed in the Accelrys Discovery studio package to generate pharmacophore model, with default parameters. The pharmacophore model was selected and then used to search the 3D structure database to identify the appropriate receptor structure.

### Protein dataset

The crystallographic structure of PI3K (α-isoform) class I lipid kinase was achieved from Brookhaven PDB (Protein Data Bank; www.rcsb.org) server^37^. We selected a catalytic subunit of α isoform (PDB ID: 4JPS) of Homo sapiens organism, solved by X-ray diffraction method at a resolution of 2.2 Å^13^.

### Pharmacokinetic properties

Drug-likeliness of 17 PBS compounds were analyzed by assessing Lipinski’s rules, Absorption, distribution, metabolism, excretion, and toxicity (ADMET) descriptors, and TOPKAT descriptors using Accelrys Discovery studio package. ADMET analyses were performed using sixdescriptors, such as absorption, solubility, CYP2D6, Plasma Protein Binding (PPB), hepatotoxicity, and AlogP98. Also, Toxicity Prediction by Komputer Assisted Technology (TOPKAT) analyses were performed using carcinogenic potency of LD50 mouse, Ames mutagenicity, Ames probability, Ames enrichment, Ames score, rat oral LD50, rat inhalational LC50, and Daphina EC50.

### Docking simulations

Binding affinities of our 17 PBS compounds against an alpha isoform of PI3-Kinase were computed using an open source software Autodock 4.2.6 version^22^. These compounds were used as ligands against PI3K- α protein receptor.

Protein structure of PI3K-α was preprocessed for docking by computing Gasteiger charges, adding hydrogens and removing water. The receptor was kept rigid while the ligands were kept flexible by setting torsion angles. Precise binding complexes of ligands and receptor molecule were obtained by setting grid maps. The grid points per map in X, Y, Z dimensions: 60 × 60 × 60 Å with 0.375 Å spacing and grid center X: −1.318, Y: −9.512, Z: 16.948, were used to cover catalytic pockets of PI3K-α. Binding conformations were estimated as per default docking parameters and Lamarckian Genetic Algorithm for ligands. For further interaction analysis, lowest binding energy conformations were plotted by LigPlot + v.1.4.5^23^.

### Zebrafish maintenance

Adult wild-type zebrafish of 4–5 months were housed in a ZebTEC Stand-Alone system (Tecniplast, Buguggiate, Varese, Italy) set at temperature 26–28 °C, pH 7.0–7.5 and conductivity 400 - 600 µS. The room photo period cycle was maintained at 14:10 h light: dark and fish were fed twice a day with freshly hatched live *Artemia* (Inve Aquaculture, Inc., Salt Lake City, USA). The experimental protocol was duly approved by the Institutional Animal Ethics Committee of CSIR-IHBT and was performed in accordance with the approved guidelines.

### Egg collection

The eggs were obtained from the induced spawning of healthy adults in separate breeding tanks. Briefly, at the end of the light cycle of the day prior to collection of eggs, two males and four females (1:2 ratio) were transferred to a breeding tank (Tecniplast, Buguggiate, Varese, Italy) separated by a transparent divider, containing system water maintained at 28.5 °C temperature. The breeding setup comprised of an internal grid bottom tank (Model: ZB10BTI) with a sliding transparent divider (Model: ZB10BTD), fitted into the external solid bottom tank (Model: ZB10BTE) covered with a transparent lid (Model: ZB10BTL). On the day of egg collection, the lid was removed after 30 min of the start of the light cycle. Healthy fertilized eggs were collected in sterile petri dishes using a pipette and were cleaned with 2–3 rounds of washing with system water. Around 50 eggs per plate were kept in a BOD incubator (Relitech, Ambala, India) at 28.5 °C. Regular water changes (twice a day) were done till 7 *dpf*.

### PTZ-induced epileptic seizures

The larvae at 7 *dpf* in different groups (*n* = 6) were pre-incubation with 3 different concentrations [0.25, 0.5 and 1 μM (selected on the basis of pilot studies)] of PBS-8 and PBS-9 for 1 h at 28.5 °C. The stock solutions of the compounds were made in pure dimethyl sulfoxide, and working dilutions were made with system water (0.01% concentration of DMSO in final solution). Following pretreatment, each larva was exposed to 8 mM of PTZ at 28.5 °C, and the induced seizures were recorded with upper cut off time of 15 min. Two separate groups of larvae (*n* = 6) were also exposed to PTZ after pre-incubation in system water and valproic acid sodium salt (3 mM in system water) that served as vehicle control and standard, respectively. Three different stages appeared in larva exposed to PTZ as, Stage I: enhanced swimming activity or hyperactivity; Stage II: circular whirlpool-like movements and; Stage III: clonic seizures with loss of posture and falling^33^. Increase in latency to clonic seizures was recorded as a parameter for anticonvulsant effect.

### Analysis of target gene expression

For gene expression studies healthy zebrafish larvae of 7 *dpf* were pre-incubated with test compounds (1 µM) for 1 h and later exposed to 8 mM PTZ solution for 15 min (3 set of larvae for each concentration, *n* = 20/set). Similar, separate sets were made that served as vehicle control (pre-incubated in system water and exposed to PTZ) and naïve control (not exposed to PTZ). At 1 h, post-PTZ exposure, total RNA was extracted from whole larvae (*n* = 20/group) using Trizol reagent (Sigma Aldrich, USA). The homogenate formed after Trizol exposure was treated with chloroform and centrifuged at 12,000 g for 15 min at 4 °C following incubation at room temperature for 5 min. The aqueous layer obtained after chloroform treatment was further subjected to 100% isopropanol to precipitate the RNA and centrifuged at 12,000 g for 10 min at 4 °C. The pellet obtained was repeatedly washed in 75% ethanol, centrifuged at 7500 g for 5 min at 4 °C, dissolved in nuclease-free water and quantified using Nanodrop ND-1000 (Thermo Scientific, USA). The total RNA obtained was treated with RNase-free DNase kit (Promega, Madison, USA) for removal of trace amounts of DNA, following which cDNA synthesis was done using high capacity cDNA-RT kit (Applied Biosystems, USA), as per manufacturers guidelines. The quantitative real-time polymerase chain reaction (qRT-PCR) analysis was done  by SYBR Green Jump start Taq Ready Mix (Sigma Aldrich, USA) on Step One Plus Real-Time PCR system (Applied Biosystems, USA) using elongation factor-1-α (*elf1α*) of zebrafish as the reference standard. The target-specific primers were designed (Table 5) using Primer Express Software 3.0 (Applied Biosystems, USA). Moreover, to reduce sampling error, the mean of each sample was considered after performing the reaction in triplicate. The annealing temperature of each gene was standardized at 55 °C, and gene expression was denoted by fold change using comparative 2^ddCT method^38^.

**Table 5:** Primer sequence of target genes.

Genes	*Forward primer (5*′ 3′)	*Reverse primer (5*′ *3*′)
*c-fos*	AACTGTCACGGCGATCTCTT	GCAGGCATGTATGGTTCAGA
*PIK3CA*	CGCAATGAGAGGATGAGCGA	ACGCTGTCACGATGGAACAA
*PIK3R1*	ACATGGCTCTGCAAGATGCT	GGAGGCATCTCGGACCAAAA
*AKT1*	TCGGCAGGTGTCTTCTCAAT	ACCCATTGCCATACCACGAG
*mTOR*	AGATCATCAACCGAGTGCGG	AGGGCACCATCCAATGTAGC
*Rps6*	TCACTCTTGTTACCGTCCTC	TGACAATGACCAAGTTGAGA
*Rps6kb1*	AAAACTCCCAAAGACTCTCC	CTAGTGGCGCACTTTTACTT
*elf1α*	GATGCACCACGAGTCTCTGA	TGATGACCTGAGCGTTGAAG

### Statistical analysis

The results of latency to clonic seizures were shown as mean ± standard error. The gene expression study results were presented as mean ± standard deviation. The statistical significance among different groups was analyzed using one-way analysis of variance followed by Tukey’s test. The results were regarded as significant at P < 0.05.
